# Suspect Screening of Chemicals in Hospital Wastewaters Using Effect-Directed Analysis Approach as Prioritization Strategy

**DOI:** 10.3390/molecules28031212

**Published:** 2023-01-26

**Authors:** Naroa Lopez-Herguedas, Leire Mijangos, Iker Alvarez-Mora, Belén González-Gaya, Teresa Uribe-Echeverria, Nestor Etxebarria, Olatz Zuloaga, Maitane Olivares, Ailette Prieto

**Affiliations:** 1 Department of Analytical Chemistry, Faculty of Science and Technology, University of the Basque Country (UPV/EHU), 48940 Leioa, Basque Country, Spain; 2 Research Centre for Experimental Marine Biology and Biotechnology (PIE), University of the Basque Country (UPV/EHU), 48620 Plentzia, Basque Country, Spain

**Keywords:** effect-directed analysis, sea urchin bioassay, suspect screening, hospital effluent, LC-q-Orbitrap

## Abstract

The increasing number of contaminants in the environment has pushed water monitoring programs to find out the most hazardous known and unknown chemicals in the environment. Sample treatment-simplification methods and non-target screening approaches can help researchers to not overlook potential chemicals present in complex aqueous samples. In this work, an effect-directed analysis (EDA) protocol using the sea urchin embryo test (SET) as a toxicological in vivo bioassay was used as simplified strategy to identify potential unknown chemicals present in a very complex aqueous matrix such as hospital effluent. The SET bioassay was used for the first time here to evaluate potential toxic fractions in hospital effluent, which were obtained after a two-step fractionation using C_18_ and aminopropyl chromatographic semi-preparative columns. The unknown compounds present in the toxic fractions were identified by means of liquid chromatography coupled to a Q Exactive Orbitrap high-resolution mass spectrometer (LC-HRMS) and using a suspect analysis approach. The results were complemented by gas chromatography-mass spectrometry analysis (GC-MS) in order to identify the widest range of chemical compounds present in the sample and the toxic fractions. Using EDA as sample treatment simplification method, the number of unknown chemicals (>446 features) detected in the raw sample was narrowed down to 94 potential toxic candidates identified in the significantly toxic fractions. Among them, the presence of 25 compounds was confirmed with available chemical standards including 14 pharmaceuticals, a personal care product, six pesticides and four industrial products. The observations found in this work emphasize the difficulties in identifying potential toxicity drivers in complex water samples, as in the case of hospital wastewater.

## 1. Introduction

The occurrence of contaminants of emerging concern (CECs), including pharmaceuticals and personal care products (PPCPs), pesticides or industrial chemicals, among other organic micropollutants, in the environment has increased during the last decades [1,2,3]. Although CECs are often found at trace levels, their daily use and continuous release into the aquatic environment are of particular concern and make the assessment of the risk that their occurrence poses to the environment and human health necessary [4].

The amount and variety of CECs is particularly large in industrial, urban and/or hospital wastewaters [4,5,6,7]. Wastewaters are often treated in wastewater treatment plants (WWTPs) but the elimination of CECs is not completely achieved and CECs as well as their transformation products can reach the aquatic environment [4,5,8]. Evidence of poor elimination of some CECs, including PPCPs, in WWTPs can be found in studies carried out in a WWTP located in the Basque Country (Galindo WWTP) [9], which receives the effluents of five hospitals and urban effluents. The non-completely eliminated compounds can reach environmental waters and they can be detected in the estuaries of the Basque Country [10,11].

This scenario is repeated over the world, so CECs, and specifically PPCPs, were introduced in the first Watch List (WL-1) of the Water Framework Directive (WFD) [12], in order to guide environmental monitoring and risk assessments. The identification of new compounds through target and non-targeted analytical strategies in various monitoring programs [13] allows the inclusion of some of them in the mentioned Watch List. In 2020, some CECs such as the antibiotics sulfamethoxazole, clindamycin and ofloxacin, the antidepressant venlafaxine and its metabolite O-desmethylvenlafaxine, the antifungals fluconazole and miconazole and the pesticides imazalil, prochloraz and tebuconazole were included in the most updated version of the Watch List, WL-3 [14]. However, the number of regulated compounds is still limited compared to the contaminants detected in aquatic environments, and consequently, substances that would pose a potential risk to the environment are overlooked [15,16,17].

In order to detect and identify as many as possible known and unknown chemicals in environmental waters, the use of suspect and non-targeted screening (SNTS) approaches using high-resolution mass spectrometry (HRMS such as Q-TOF/Q-Orbitrap) coupled to liquid or gas chromatography (LC or GC) has been seen in most scientific works [18,19,20,21,22,23]. However, regardless of the data acquisition method used, data processing is still one of the biggest bottlenecks of the full analytical process and the source of endless discussions [18], especially when the analysis of very complex water samples (e.g., samples containing a huge cocktail of unknown chemicals and a large amount of organic matter) is required. Hence, prioritization strategies based on feature data such as peak areas, blank/sample ratio, detection frequency, time trends, contaminant mode of action, toxicity, and source-related suspect screening, to name a few, are used in SNTS [18,24]. Effect-directed analysis (EDA), which aims to elucidate cause-effect relationships, is also a potential tool to handle the problem [17,25].

EDA is a prioritization tool based on a sequential reduction in the complexity of a sample via fractionation, and effect-based monitoring with advanced large-scale chemical profiling (suspect and non-target screening) to isolate and identify toxicity driver compounds in a few active fractions. In EDA protocols, in vitro cell-based bioassays are the preferred tools to determine toxicity due to their feasibility and sample analysis high throughput [26,27,28]. However, in trying to get more accurate estimations of toxicologically relevant effects, the use of in vivo bioassays based on different organisms such as larvae and embryo assays is increasing [29,30,31]. In vivo bioassays allow for the more accurate determination of effects when a general toxicological answer is evaluated at organism level (e.g., zebrafish [29] at early life stages, mudsnails [31], mussels [32] or sea urchins [30]) and, when local organisms are used, the contamination of a specific area can be more precisely determined. In this sense, the sea urchin (*Paracentrotus lividus*) embryo test (SET) has arisen as a key in vivo assay for coastal marine/estuarine ecosystems [33]. Their use has been included in the European Union Reference Laboratory for Alternatives to Animal Testing (*sea urchin embryo test*, SET), and it has been standardized by several national environmental agencies [34,35,36]. Indeed, many studies have proven the sensitivity of sea urchin organisms to emerging contaminant presence [37,38] and the suitability of SET bioassay in EDA strategies [30]. Despite its demonstrated potential as a standardizable bioassay [38,39], studies reporting the use of sea urchins in EDA are scarce [30,40].

Within this scenario, the present work aimed to use a previously validated EDA methodology [34], using, for the first time, the sea urchin embryo in-vivo toxicological test (SET) as sample treatment simplification strategy to unravel potential unknown chemicals present in a very complex aqueous matrix such as hospital effluent.

## 2. Results and Discussion

### 2.1. Quality Assurance and Quality Control

In this work, a previously optimized and validated fractionation protocol for EDA was used [41] (see Table S1 in Supplementary Materials). Regarding the SET bioassays quality parameters, procedure blank samples (LV-B) and test control samples were prepared. On the one hand, solvent control samples (i.e., filtered seawater (CFSW) and DMSO (CDMSO)) were prepared and analyzed in parallel with the samples (see Section 3.3.2). Solvent control samples did not exhibit any effects on the two toxicological endpoints tested: sea urchin size increase (SI, %) and larvae morphology (IT) (Figure S2). Three replicates of blank samples (LV-B) were fractionated to detect contamination during the fractionation step (LV-B-R). None of the tested blanks induced any effect on the SI (%) of the exposed larvae (Figure S2) and neither carryover nor contamination was detected in the chemical analyses.

### 2.2. Hospital Effluent Toxicity Evaluation and Identification of Toxic Fractions through EDA

**Figure 1:**
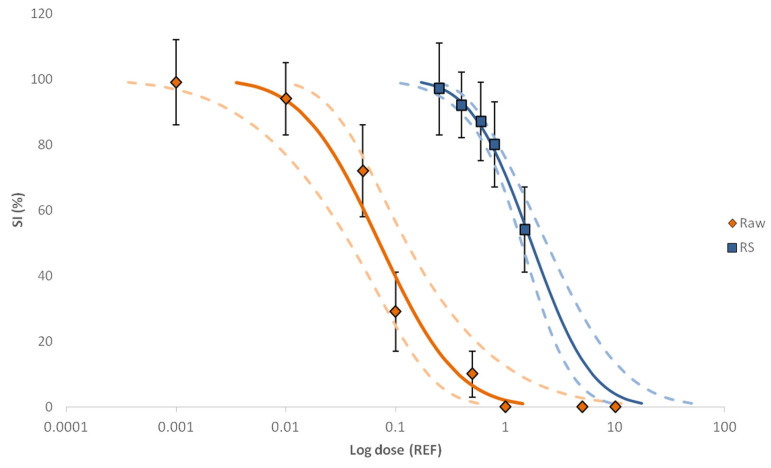
The log dose–response curves of raw (hospital effluent) and RS samples obtained with SI endpoint (%). Continuous lines show the effect concentration (EC) fit values obtained with PROBIT and dashed lines indicate the confidence level (95%). Error bars correspond to the standard deviation (SD) of the three replicates.

The level of toxicity of the control samples and the raw sample of the hospital effluent was tested in triplicate at eight concentration levels in the range of REF 0.001–10. Based on a PROBIT-modelled log dose–response curve (i.e., size increase vs. REFs), the raw sample showed embryo growth inhibition and skeleton malformation activity within the concentration range tested, except the two lower ones (REF 0.001 and 0.01) where four-arm pluteus stage was reached and were not significantly different from the controls (ρ < 0.05, Kruskal–Wallis) (see Figure 1). Growth inhibition was observed at concentration levels equal to REF 0.1 while concentration values above REF 1 caused full inhibition. In this way, the EC_50_ value was set at REF 0.07, and the calculated TU_bio_ value was 14.1 at that EC_50_ level. The IT values were also calculated for different REFs (Figure S3A,B). Based on those values, a significant toxicity increase was observed in the range of 0.05–0.1 REF, and the maximum IT values (IT = 3) were reached at REFs ≥ 0.5.

The toxicity of the RS was tested at concentrations between REFs 0.25–1.5 to determine any total toxicity loss during the fractionation step. Based on the log–dose–SI response curve (see Figure 1), a bioactivity loss was observed for the recombined raw sample (RS) (EC_50_ = 1.73). Since the quality parameters of the EDA fractionation are acceptable [41], the decrease in the bioactivity can be attributed to (1) the type of bioassay used, as it is a phenomenon often found when bioassays indicating adaptive stress responses or apical endpoints are used [42], or (2) the removal of the organic matter present in the raw sample due to fractionation [43].

**Figure 2:**
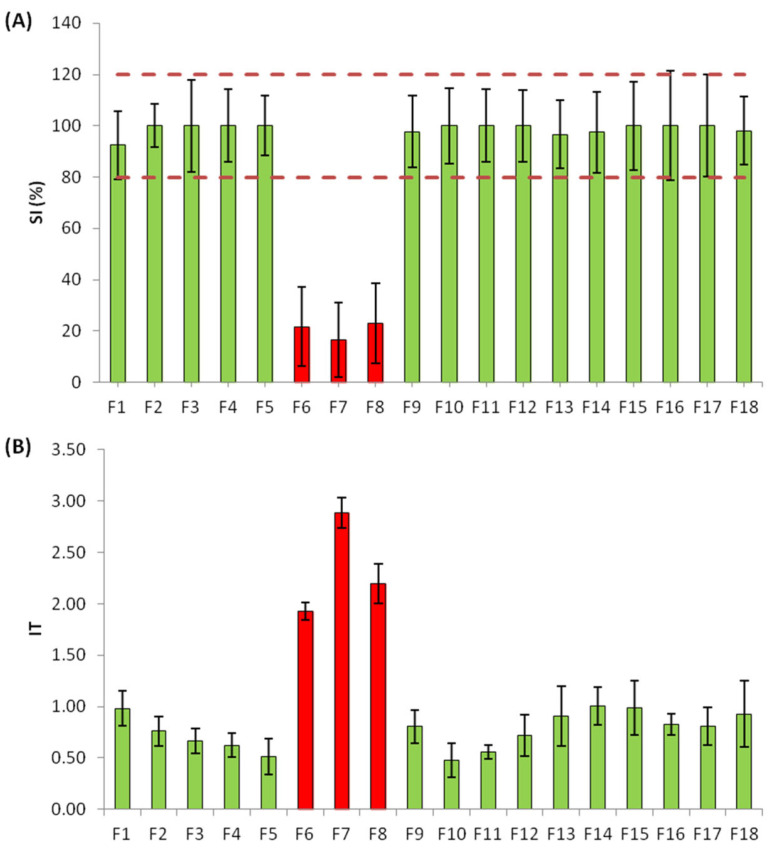
Toxicity of the fractions obtained in the first fractionation at REF 0.3. (**A**) Size increase (%) response of the fractions and (**B**) index of toxicity (IT) response of the fractions based on larvae malformations. Green colored bars refer to non-toxic fractions, while red bars refer to the identified toxic fractions. Error bars correspond to the standard deviation (SD) of the three replicates. Red dotted lines represent normal larvae SI (%) range (80–120%).

A toxicity screening of all 18 primary fractions was performed at REF 0.07 (equivalent to EC_50_ of the raw sample) and REF 0.3 (equivalent to EC_85_ of the raw sample). Toxic effects measured as SI% and IT were only observed in three of the fractions tested (F6, F7 and F8) at REF 0.3 (ρ_value_ < 0.05, Kruskal–Wallis) (see Figure 2A,B for REF 0.3 and Figure S4 for SI (%) at REF 0.07).

In order to reduce the sample complexity to identify chemicals, the three toxicologically active fractions (i.e., F6, F7 and F8) were combined (ΣF) (EC_50_ ≈ REF 0.5) and fractionated again, obtaining 16 secondary fractions (see Section S2.2 in Supplementary Materials). Secondary fractions were tested at REF 0.5 considering the better isolation of the toxic compounds when fractionating, and therefore, the need for higher REFs for toxicity detection. However, none of the fractions obtained after the second fractionation stood out as toxic, suggesting a spread of the toxicity driver compounds over many fractions, below the detection limit (ρ_value_ < 0.05, Kruskal–Wallis) (Figure S5).

**Figure 3:**
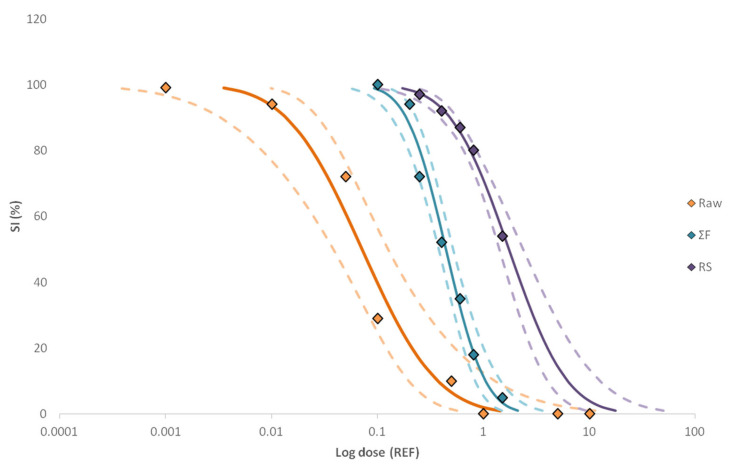
The log dose–response curves of the active samples and fractions (Raw, RS, ΣF and ΣF-RS) obtained with the size increase (%) endpoint. Straight lines show the EC fit values and dashed lines indicate the confidence level (95%).

Comparisons of dose–response relationships between the raw sample, RS and ΣF revealed that the raw sample was the most toxicologically active, being significantly different (*p*-value < 0.05, 95% confidence level) (Figure 3). Furthermore, the overlapping intervals of those may indicate that further isolation of the key toxicants and/or organic matter removal was achieved with the second fractionation, even if the toxicity was not recovered in a single fraction. Moreover, the EC_50_ and TU_bio_ values given in Table 1 sustain the significant differences between the raw sample and the toxicologically active fractions since about 16% of the hospital effluent bioactivity was recovered in ΣF (TU_bio_ = 2.3), while the remaining unexplained activity was probably spread over the other fractions [30,42].

### 2.3. Identification of Unknown Compounds

Hospital effluent raw sample and all toxic fraction extracts were analyzed through LC-qOrbitrap and GC-MS to identify as many as possible unknown organic compounds. Concerning LC-qOrbitrap, in total, 446 features were among the compounds belonging to the relevant suspect lists in the raw sample, but only 94 features were present in the toxic fractions (see Table S2 in Supplementary Materials). Concretely, based on the Schymanski classification [44,45], the presence of 23 compounds was confirmed (level 1), 29 compounds were tentatively identified as probable structures (Level 2a or 2b) and 42 as tentative candidates (Levels 3 and 4) (see Table S2 in Supplementary Materials for the full list of identified features). In Figure S6, the extracted ion chromatogram (A) and the mass spectra similarity between the experimental and the mzCloud library (B) of bisoprolol are included as an example of level 1 compound identification.

The compounds identified as level 1 were quantified in the raw hospital effluent sample (see Table 2). Among the compounds identified as level 1, 14 pharmaceuticals were detected at concentration levels between 3 ng/L (propranolol) and 37 ng/mL (furosemide). In addition to pharmaceuticals, seven pesticides (one herbicide, an insecticide and four fungicides), an industrial product, a preservative and a flame retardant were also quantified (see Table 2).

Concerning the analysis by GC-MS, the presence of two compounds (see Table S2), diethyl phthalate (DEP) and 2,4-di-tert-butylphenol (2,4-DBT), was confirmed and they were also quantified in the hospital effluent (see Table 2).

Several worldwide studies reported the presence of some of the detected compounds in hospital effluents. Santos et al. [7] reported the occurrence of the pharmaceuticals furosemide, ofloxacin, propranolol, sulfamethoxazole and verapamil in four different hospitals located in Coimbra (Portugal) at similar concentration levels to those determined in this work. Although at lower concentration levels, the compounds diphenhydramine, furosemide, methylparaben, omeprazole, primidone, propranolol, sulfamethoxazole and verapamil were also detected in hospital effluents monitored in New York [6]. The presence of fluconazole, furosemide and clozapine was also reported in a study carried out in Uppsala (Sweden) where hospital effluent samples were analyzed [46]. On a national scale, the occurrence of omeprazole, propranolol and sulfamethoxazole was observed in hospital effluent (Galdakao, Biscay) but at higher concentrations than the ones determined in this study. To the best of our knowledge, the occurrence of the remaining compounds detected in this study has not been reported in any other hospital effluent. Nevertheless, the occurrence of such compounds can be expected. For example, although the presence of the pesticides acetamiprid or spiroxamine was not reported in other hospital effluents, pesticides such as atrazine or metalaxyl were found in a hospital effluent located in Beijing, China [47]. The presence of OBT, the hydroxylated metabolite of benzothiazole, can be explained due to the extensive use of benzothiazole compounds (e.g., corrosion inhibitors, ultraviolet light stabilizers in textiles and plastics, food flavoring agents and pharmaceuticals) which can end up not only in environmental compartments [10,11,48] but also ingested by humans and then excreted as OBT via urine [49]. 2,4-DBT, DEP and TEP are widely used plasticizers (e.g., in PVC plastics) and have already been detected in several aqueous environmental samples (e.g., in sewage waters [4,50,51], surface waters [4,51,52] and drinking waters [50]); hence, they are likely to be found in hospital effluent.

#### Assessment of Potential Toxicity of Identified Compounds

The potential toxicity of all the compounds identified as level 1 was assessed (Table S3) at the concentration level quantified in the raw sample. At those conditions, none of the candidates showed any measurable effect at REF 50 (see Figure S7A,B in Supplementary Materials), concluding that, even if they were identified in the toxic fractions, none of them could be identified as responsible for the sample toxicity at tested conditions. Due to the lack of available ecotoxicological data for sea urchins, an additional toxicity assessment of the candidates was based on the acute toxicity values (EC_50_ and LC_50_) reported in the literature for fish and invertebrates (Table S3). The effects of the individual compounds observed in this study are in line with the results reported in other studies [53,54,55,56,57,58,59,60,61,62,63,64,65,66,67,68,69], online available databases (Pesticides Properties Database, PPDB, http://sitem.herts.ac.uk/aeru/ppdb/, accessed on 2 May 2022) and estimated values with Ecological Structure–Activity Relationship (ECOSAR) modelling (version 2.0, downloaded from https://www.epa.gov/tsca-screening-tools/ecological-structure-activity-relationships-ecosar-predictive-model, accessed on 29 April 2022) since the EC_50_ and LC_50_ values are much higher than the calculated concentrations of the compounds. In any case, as can be observed in Table S3, EDDP (0.000032 mg/L) was ranked at the top of the list of toxicants for invertebrates, followed by TEP (0.02 mg/L) and 2,4-DBT (0.281 mg/L), fenpropidin (0.54 mg/L), isoproturon (0.58 mg/L), eprosartan (0.593 mg/L) and propranolol (0.8 mg/L) to a lesser extent. As for fish, the lowest values of LC_50_ were compiled for eprosartan, 2,4-DBT and EDDP (0.011, 0.144 and 0.43 mg/L, respectively). Moreover, a previous EDA study to address the impact of WWTP effluents on coastal ecosystems through SET reported similar levels of fenpropidin (23 ng/L) and an experimental EC_50_ value of 560,000 ng/L explaining <0.1% of the whole toxicity [30]. Prieto-Amador performed an ecotoxicological assessment of several phthalates in the sea urchin *P. lividus*, in which an experimental EC_50_ value of 758.58 µg/L for DEP was reported [70], thus evidencing its low contribution to the toxicity detected in this study. On the other hand, Ribeiro et al. reported a significant increase in the percentage of total sea urchin abnormalities over 125 µg/L of propranolol [71].

The toxicity of the chemical mixture was also tested, but as occurred when the individual chemicals were tested, the mixture did not show toxicity up to a concentration 1000-fold above the original hospital effluent concentrations (see Figure S8).

Similar to the observations found in this work regarding unexplained toxicity, several studies integrating biological and chemical analysis showed unexplained effects for a variety of specific and non-specific bioassays [72,73,74,75,76]. In the case of non-specific bioassays, as is the case with SET, the elucidation of key toxic compounds can be hindered due to the alteration in many different pathways of the organism in a non-targeted way [72,76]. Moreover, the existence of several toxicity mechanisms/pathways leads to the consideration of possible interactions between the compounds present in the sample (e.g., synergism and/or antagonism) that could contribute considerably to the observed toxicity. For instance, Neale et al. [72] used the non-specific FET test to cover a wider range of endpoints to determine the effects on the human-impacted Danube River (Serbia), but the identified compounds could explain less than 0.2% of the toxicological effects on zebrafish. However, to a lesser extent, examples of unexplained toxicity can also be found in the literature, even when using specific bioassays. When using specific bioassays, the observed effects can be associated with specific modes of action (e.g., binding estrogen or androgen nuclear receptor or photosystem II), and the active compounds should share structural similarities. However, some examples in the literature pointed out the potential presence of non-identified active chemicals in different water ecosystems (e.g., wastewater effluent or surface waters) [72,73,74], even if, for example, activation of the NFκB mediated inflammatory pathway was observed. In the same way, Kienle et al. [75] also reported a substantial difference between the biological effects and chemical results for the acetylcholinesterase inhibition bioassay on WWTP effluent samples, which could be related to the content of the dissolved organic carbon of the sample.

## 3. Experimental Section

### 3.1. Reagents and Materials

All standard chemicals and materials used in this work are described in Lopez-Herguedas et al. [41].

### 3.2. Sampling

The sampling site and sampling method are thoroughly described elsewhere [41] and in the supplementary material (Section S2.1). Briefly, 45 L of a 24 h composite water sample was collected from the main discharging sewage drain of a hospital (holding > 800 beds and offering > 13 specialties) (Biscay, Basque Country, Spain) using an on-site large-volume solid phase extraction (LV-SPE (MAXX Mess-u. Probenahmetechnik GmbH, Rangendingen, Germany). For water sample analysis, SPE cartridges were eluted following the procedure proposed by Välitalio et al. [77] (see Supplementary Materials, Section S2.1). In parallel, and for quality control assurance, 5 L of UHPLC-MS grade water mineralized with analytical grade sodium chloride (0.1%, Merck, Darmstadt, Germany) was pumped 9 times through an LV-SPE system in order to have a blank sample (LV-B) The hospital effluent sample and blank sample were enriched 250 times (EF 250).

### 3.3. Effect-Directed Analysis (EDA)

**Figure 4:**
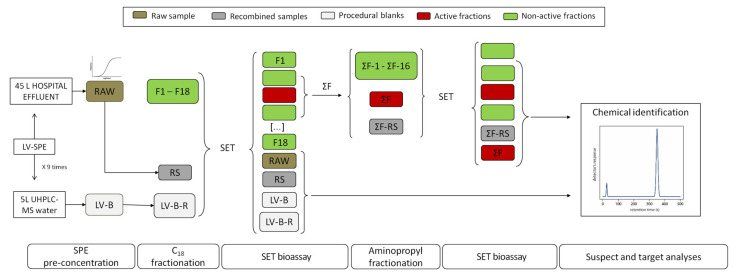
Workflow of EDA with orthogonal fractionation. Solid phase extraction (SPE), sea urchin embryo test (SET) bioassay, blank samples (B). For the first fractionation: fractions (F1–18), raw sample (RAW), recombined sample (RS), pool of the three active fractions (ΣF); for the second fractionation: fractions (ΣF-1–16) and recombined sample (ΣF-RS).

EDA was used as a prioritization tool before chemical identification. To that aim, the preconcentrated hospital and blank samples were submitted to the EDA protocol described in a previous work of the research group [41] (see details in Figure 4 and Section S2 in Supplementary Materials). Briefly, the preconcentrated hospital effluent water sample (raw) and blank sample (LV-B) were fractionated by a 2-step procedure. The first fractionation of raw and LV-B was conducted using a C_18_ semi-preparative column and, the toxic fractions were fractionated again using an aminopropyl semi-preparative column. A replicate of the raw sample was fractionated again and the fractions obtained in each fractionation were recombined (RS and ΣF-RS for the first and second fractionation, respectively) to assure that no analyte or bioactivity losses occurred during fractionation. The bioactivity of the raw sample (raw), recombined samples (RS and ΣF-RS), individual fractions as well as procedural (LV-B) and recombined blanks (LV-B-R) was tested using the SET bioassay (see Section 3.3.1.). In order to test the whole bioactivity in a unique fraction, the active fractions found in the first fractionation were combined (ΣF). Chemical analysis was limited to biologically active and neighboring fractions, the RS and ΣF-RS, and raw and blank samples (see Section 3.3.2).

#### 3.3.1. Sea Urchin Embryo Test (SET)

Adult sea urchins (*Paracentrotus lividus*) were collected from an intertidal area of Armintza (43.43347 N, 2.89889 W, Basque Country) and maintained in an aquarium at the Research Centre for Experimental Marine Biology and Biotechnology, Plentzia Marine Station (PIE, Basque Country). Seawater tanks were maintained at 15 ± 1 °C with a natural photoperiod (12:12 h) and a continuous aeration and filtration water pumping system. Every two days, sea urchins were fed with macroalgae gathered in their sampling area and the dregs were siphoned. SET bioassays were performed following the procedure described by Saco-Álvarez et al. [78] detailed in the Supplementary Materials (see Section S2.3). Briefly, raw sample extracts and SPE eluted extracts were evaporated up to dryness under a gentle stream of N_2_ at 40 °C on a turbovap system and re-dissolved using filtered seawater (FSW, 0.2 µm) containing 0.1% of DMSO (*v*/*v*). Three mL of the diluted samples (n = 3) were placed in glass vials (20 mL) with the fertilized eggs at a 40 eggs/mL concentration. Likewise, FSW (CFSW) and FSW with 0.1% of DMSO (*v*/*v*) (CDMSO) were also included in the test batch as solvent controls, and a copper (II) chloride solution (0.01–5 mg/L) was used as a positive quality control sample. Fertilized eggs (egg size at t = 0) and samples after the incubation stage (20 °C, 48 h in darkness) were fixed with one drop of 40% formalin.

Toxicity assessment was carried out by measuring two different sublethal points: (1) the index of toxicity (IT), where individual embryos were categorized for their malformation level according to Carballeira et al. [79], and (2) the size increase (SI) in the larvae recorded according to Saco-Álvarez et al. [78] (detailed in Supplementary Materials, Section S2.3, Figure S1). Measurements were performed via an inverted microscope (Nikon Eclipse Ti-S) coupled to an electronic camera using NIS-Elements image analysis software v4.30 (Nikon Instruments BV, Europe). The hospital effluent (raw sample) was tested in dilution series (REF 0.001–0.01–0.05–0.1–0.5–1–5–10) to build the dose-response curve from which to derive the concentration value that causes effects in at least half of the population (effective concentration, EC_50_).

Data treatment and statistical analyses were processed with the SPSS Statistics 23 package (v17, IBM SPSS), using data corrected by the control response. As the data set was not normally distributed (Shapiro–Wilk test), the non-parametric Kruskal–Wallis test was performed to compare treatments. The PROBIT model was used to determine the EC_i_ values with 95% confidence limits.

#### 3.3.2. Chemical Analysis

Samples and fractions exhibiting significant toxicity in the SET bioassay were analyzed using LC-HRMS and GC-MS analyses and the methodologies described elsewhere [4,80] (see Supplementary Materials, Section S2.4). Blank samples (LV-B, LV-B-R) were also analyzed simultaneously, to avoid false positive results.

Concretely, the LC-HRMS measurements were run in a Thermo Scientific Dionex UltiMate 3000 UHPLC coupled to a Thermo Scientific™ Q Exactive™ Focus quadrupole-Orbitrap mass spectrometer (UHPLC-q-Orbitrap) equipped with a heated ESI source (HESI, Thermo-Fisher Scientific, CA, USA). The LC-qOrbitrap measurements were acquired in negative and positive ionization in the Full scan—data-dependent MS2 (Full MS-ddMS2) discovery acquisition mode in the *m/z* 70–1050 Da, which allowed us to identify compounds through suspect screening analysis as well as multi-target analysis. Suspect analysis was performed employing Compound Discoverer 3.1 software (CD, Thermo-Fischer Scientific) following the workflow described by González-Gaya et al. [41], with some modifications (detailed in Supplementary Materials, Section S2.4.1). Regarding the multi-targeted analysis of the 187 target compounds available at the laboratory (see details in Table S1 in Supplementary Materials), the compounds were identified and quantified using TraceFinder 4.1 as detailed in the Supplementary Materials (see Section S2.4). Instrumental characteristics including ionization mode, retention time (R_t_), and instrumental limits of quantification (LOQ_instrumental_) and identification (LOI_instrumental_) of the 187 target compounds are detailed in Table S1.

Regarding GC-MS analyses, an Agilent 6890 gas chromatograph coupled to an Agilent 5975 mass spectrometer system (Agilent Technologies, Palo Alto, CA, USA) (see Supplementary Materials, Section S2.4.2) was used. The measurements in SCAN mode allowed the identification of unknown non-polar compounds from the comparison of experimental mass spectra with the ones available in the NIST library, and the identified compounds were afterwards confirmed using standards.

#### 3.3.3. Potential Toxicity Assessment of Detected Compounds

The potential toxicity of compounds identified in the toxic fractions of the hospital effluents was evaluated through the estimation of toxic units (TU). TU values were calculated as described in a previously published EDA study [30]. Concretely, chemically based TU values (TU_chem_) were calculated from the sum of the concentration of compounds identified in the toxic fractions (C_i_) normalized to individual 50% effect concentrations (EC_50(i)_) (see Equation (1)), for comparison with biologically derived TU (TU_bio_) (Equation (2)). Additionally, an artificial mixture containing the compounds identified in the toxic fractions was prepared by fortifying filtered seawater at the concentration levels at which contaminants were quantified in the hospital effluent (concentration range of 10^−3^–10^8^ ng/L). Artificial TU (TU_artificial mixture_) was determined using Equation (3).

(1) $$TU_{chem}=\overset{n}{\underset{i=1}{\sum }}\frac{C_{i}}{EC_{50\left(i\right)}}$$

(2) $$TU_{bio}=\frac{1}{EC_{50\left(sample\right)}}$$

(3) $$TU_{artificial\;mixture}=\frac{\sum^{\;}C_{i}}{EC_{50\left(mixture\right)}}$$

For the TU determination, SETApp [40] was used to automatically measure the two endpoints of the SET (i.e., IT and SI). Briefly, larvae were transferred to a 24-well microplate at 48 h post-fertilization, and an image set of each well was obtained using the automatic image reader Cytation 5 (BIOTEK). Images were loaded in the SETApp to automatically detect, isolate and measure all larvae, and determine the malformation levels according to the work of Carballeira et al. [79].

## 4. Conclusions

The EDA approach enabled the identification of potentially toxic chemicals among all the hundreds of chemicals that are present in a complex water sample, as is the case with hospital effluent. Concretely, the fractionation of the sample and toxicity testing using SET bioassays enabled the identification of three potential toxic fractions among the 18 fractions into which the raw sample was firstly divided. In this way, EDA was used in this work as a prioritization strategy in order to identify as many as possible chemicals that may be responsible for the raw sample toxicity. Suspect screening of the toxic fractions using LC-qOrbitrap and GC-MS allowed the identification of 25 potentially toxic unknown compounds including different pharmaceuticals (bisoprolol, clozapine, cyclophosphamide, diphenhydramine, eprosartan, fluconazole, furosemide, ofloxacin, omeprazole, pentoxifylline, propranolol, primidone, sulfamethoxazole, verapamil), pesticides (acetamiprid, EDDP, fenpropidin, imazalil, isoproturon, spiroxamine), industrial compounds (2,4-DBT, OBT, DEP, TEP) and a preservative (methylparaben), among other tentatively identified substances. However, sample toxicity was neither attributed to any particular compound as a predominant driver nor confirmed as the result of a combined effect of the cocktail of chemicals present in the hospital effluent.

The discrepancies observed between analytical and bioanalytical contamination suggest that even though efforts have been made to identify unknown toxic compounds, there are more unidentified compounds at concentration levels below the limits of detection and/or not detectable by the chemical analyses used in this work. In this way, these results support the need to improve the detection limits of analytical techniques to facilitate the identification of undetected/unknown contaminants. On the other hand, the lack of ecotoxicological data on detected contaminants for sea urchins should also be highlighted. As a model organism for assessing the ecological status of coastal ecosystems, it would be necessary to broaden the knowledge of the effects in sea urchins of these pollutants, considering their ubiquitous reported presence in the environment. Furthermore, the observations found in this work highlight the importance of further investigating the different toxicity pathways as it could help us understand how contaminants interfere in different non-specific bioassays as well as lead to the presence of mixture effects.

## Figures and Tables

**Table 1:** Toxic units (TU_bio_) for half effective concentrations (EC_50_) obtained with size increase (SI) endpoint with their confidence level (95%) for the bioactive samples and fractions.

Sample	Size Increase (SI)	
	EC_50_	TU_bio_
Raw	0.071	14.1
	(0.038–0.125)	(8.0–26.3)
RS	1.733	0.6
	(1.410–2.371)	(0.4–0.7)
∑F	0.442	2.3
	(0.380–0.517)	(1.9–2.6)

**Table 2:** Concentrations (ng/L) of potentially toxic compounds detected in the raw sample. Their application and mechanisms of action (MoAs) are also included.

Compound	Raw (ng/L)	Application	MoA
2,4-Di-tert-butylphenol *	36,245	Industrial product/Plasticizer	Unknown
2-Hydroxybenzothiazole	914	Industrial product/Chemical manufacturing	Unknown
Acetamiprid	24	Pesticide/Insecticide	Nicotinic acetylcholine receptor (nAChR)
Bisoprolol	1051	Pharmaceutical/Antihypertensive	β-blocker
Clozapine	8	Pharmaceutical/Antipsychotic	Neurotransmitter receptor blocker
Cyclophosphamide	138	Pharmaceutical/Antineoplastic	DNA replication and protein synthesis inhibitor
Diethyl phthalate *	63,097	Industrial product/Plasticizer	Endocrine disruptor
Diphenhydramine	3	Pharmaceutical/Antihistamine	Histamine H1 receptor
Ediphenfos	8	Pesticide/Fungicide	Acetylcholinesterase and phospholipid biosynthesis inhibitor
Eprosartan	602	Pharmaceutical/Antihypertensive	Angiotensin receptor or enzyme
Fenpropidin	20	Pesticide/Fungicide	Sterol biosynthesis inhibition
Fluconazole	4138	Pharmaceutical/Antifungal	Sterol biosynthesis inhibition
Furosemide	37,232	Pharmaceutical/Diuretic	Ion channel modulation
Imazalil	13	Pesticide/Fungicide	Sterol biosynthesis inhibition
Isoproturon	7	Pesticide/Herbicide	Photosynthesis inhibition
Methylparaben	15,614	Personal care product/Preservative	DNA, RNA and enzymes synthesis inhibitor
Ofloxacin	6154	Pharmaceutical/Antibiotic	Nucleic acid biosynthesis
Omeprazole	923	Pharmaceutical/Gastric disorders	Proton pump inhibition
Pentoxifylline	98	Pharmaceutical/Anticoagulant	Signal transduction/Erythrocyte phosphodiesterase
Primidone	6818	Pharmaceutical/Antiepileptic	Neuroactive/GABA receptor
Propranolol	3	Pharmaceutical/Antihypertensive	β-blocker
Spiroxamine	211	Pesticide/Fungicide	Sterol biosynthesis inhibition
Sulfamethoxazole	2133	Pharmaceutical/Antibiotic	Dihydropteroate synthesis
Triethylphosphate	15	Industrial product/Flame retardant	Enzyme inhibition
Verapamil	10	Pharmaceutical/Antihypertensive	Calcium ion channel modulation

* quantified by GC-MS analysis.

## Data Availability

Data will be made available on request.

## Supplementary Materials

The following supporting information can be downloaded at: https://www.mdpi.com/article/10.3390/molecules28031212/s1, Figure S1: Examples of how to measure the maximum dimension according to Saco-Álvarez [43] in P. lividus at fertilized eggs (A) and completely developed larvae at 4 arm plateaus stage (B); Figure S2: Toxicity of the tested procedural blanks (LV-Blank and LV-R-Blank) and the difference with the Raw and RS samples considering only the SI (%) endpoint. A comparison with solvent controls (CFSW and CDMSO) is also included. Green coloured bars refer to non-toxic samples/concentrations, while red bars refer to toxic samples/concentrations. Error bars correspond to the standard deviation (SD) of the three replicates; Figure S3: Toxicity level measured for each REF of the hospital effluent according to larvae malformation endpoint: (A) Calculated Index of Toxicity (IT) for each REF of the sample. Colour bar shift goes from non-toxic REFs (green) to REFs with higher toxicity (red). Error bars correspond to the standard deviation (SD) of the three replicates. (B) Malformation of the observed larvae in each REF of the sample; Figure S4: Size increase (%) response of the fractions from the first fractionation at REF 0.07 (EC50 of the raw sample). Green coloured bars refer to non-toxic fractions. Error bars correspond to the standard deviation (SD) of the three replicates; Figure S5: Size increase (%) response of the fractions from the second fractionation at REF 0.5 (EC50 of the ΣF). Green coloured bars refer to non-toxic fractions. Error bars correspond to the standard deviation (SD) of the three replicates; Figure S6: Example of the confirmation via suspect screening of the pharmaceutical bisoprolol (level 1), top priority compound found in the active fractions F6 and F7. (A) Extracted ion chromatogram and (B) experimental and mzCloud library mass spectra similarity; Figure S7: Results of sea urchin P. lividus larvae exposed to REF 50 of individual compounds detected in the toxic fractions using the SETApp [40]: (A) Size increase (%) response. Error bars correspond to the standard deviation (SD) of the three replicates. (B) IT values based on malformation of the observed larvae; Figure S8. Results of sea urchin P. lividus larvae exposed to different concentrations of the artificial mixture containing toxicity driver candidates using the SETApp [40]: (A) Size increase (%) response. Error bars correspond to the standard deviation (SD) of the three replicates. (B) IT values based on malformation of the observed larvae. (C) Malformation of the observed larvae: (1) REF 0.01, (2) REF 0.1, (3) REF 1, (4) REF 10, (5) REF 50 and (6) REF 1000; Table S1: The electrospray ionization (ESI) mode, m/z, retention times (tR) and instrumental limits of identification (LOI instrumental, ng/mL) and quantification (LOQ instrumental, ng/mL) for the target compounds classified according its application, CAS number, molecular formula, InChIKey and the mass analyser used in their analysis; Table S2: Formula, candidates, application, molecular weight (MW) and parameters to determine levels of identification of the compounds classified according to Schymanski criteria [49] with a mzCloud best match >70% in both ionization modes. Features detected in the active fractions (F6, F7 and F8) are also indicated; Table S3: Acute toxicity data of the potential toxic candidates for fish and invertebrates. Compounds structure is also included. Reference [81] was cited in the Supplementary Materials.

## Sample Availability

Samples of the compounds are available from the authors.
