# Supplementary material for: Suspect Screening of Chemicals in Hospital Wastewaters Using Effect-Directed Analysis Approach as Prioritization Strategy
Source: Molecules. 2023 Jan 26;28(3):1212. doi: 10.3390/molecules28031212 (PMC9921743; doi:10.3390/molecules28031212)
Supplement: Supplementary file 1 [file molecules-28-01212-s001.zip › molecules-2147106-supplementary.pdf]

SUPPLEMENTARY INFORMATION

Suspect Screening of Chemicals in Hospital Wastewaters Using Effect-Directed Analysis Approach as Prioritization Strategy

Naroa Lopez-Herguedas [1,2,*], Leire Mijangos [1,2], Iker Alvarez-Mora [1,2], Belén González-Gaya [1,2], Teresa Uribe-Echeverria [2], Nestor Etxebarria [1,2], Olatz Zuloaga [1,2], Maitane Olivares [1,2] and Ailette Prieto [1,2,*]

1 Department of Analytical Chemistry, Faculty of Science and Technology, University of the Basque Country (UPV/EHU), 48940 Leioa, Basque Country, Spain
2 Research Centre for Experimental Marine Biology and Biotechnology (PIE), University of the Basque Country (UPV/EHU), 48620 Plentzia, Basque Country, Spain
* Correspondence: naroa.lopez@ehu.eus (N.L.-H.); ailette.prieto@ehu.eus (A.P.)

Table of Contents

S1. REAGENTS AND MATERIALS 2

S2. EFFECT-DIRECTED ANALYSIS 3

S2.1. SAMPLING AND SAMPLE TREATMENT 3
S2.2. FRACTIONATION AND SOLID-PHASE EXTRACTION (SPE) 4
S2.3. SEA URCHIN EMBRYO TEST (SET) BIOASSAY 5
FIGURE S1. EXAMPLES OF HOW TO MEASURE THE MAXIMUM DIMENSION ACCORDING TO SACO-ÁLVAREZ [78] IN *P. LIVIDUS* AT FERTILIZED EGGS (A) AND COMPLETELY DEVELOPED LARVAE AT 4 ARM PLATEAUS STAGE (B). 6
S2.4. CHEMICAL ANALYSIS AND DATA TREATMENT 6

S3. EDA-QUALITY CONTROL 8

FIGURE S2. TOXICITY OF THE TESTED PROCEDURAL BLANKS (LV-BLANK AND LV-R-BLANK) AND THE DIFFERENCE WITH THE RAW AND RS SAMPLES CONSIDERING ONLY THE SI (%) ENDPOINT. A COMPARISON WITH SOLVENT CONTROLS (CFSW AND CDMSO) IS ALSO INCLUDED. GREEN COLOURED BARS REFER TO NON-TOXIC SAMPLES/CONCENTRATIONS, WHILE RED BARS REFER TO TOXIC SAMPLES/CONCENTRATIONS. ERROR BARS CORRESPOND TO THE STANDARD DEVIATION (SD) OF THE THREE REPLICATES 8

S4. EDA-SET 9

FIGURE S3. TOXICITY LEVEL MEASURED FOR EACH REF OF THE HOSPITAL EFFLUENT ACCORDING TO LARVAE MALFORMATION ENDPOINT: A) CALCULATED INDEX OF

TOXICITY (IT) FOR EACH REF OF THE SAMPLE. COLOUR BAR SHIFT GOES FROM NON-TOXIC REFS (GREEN) TO REFS WITH HIGHER TOXICITY (RED). ERROR BARS CORRESPOND TO THE STANDARD DEVIATION (SD) OF THE THREE REPLICATES. B) MALFORMATION OF THE OBSERVED LARVAE IN EACH REF OF THE SAMPLE. 9
FIGURE S4. SIZE INCREASE (%) RESPONSE OF THE FRACTIONS FROM THE FIRST FRACTIONATION AT REF 0.07 (EC_{50} OF THE RAW SAMPLE). GREEN COLOURED BARS REFER TO NON-TOXIC FRACTIONS. ERROR BARS CORRESPOND TO THE STANDARD DEVIATION (SD) OF THE THREE REPLICATES........ 10
FIGURE S5. SIZE INCREASE (%) RESPONSE OF THE FRACTIONS FROM THE SECOND FRACTIONATION AT REF 0.5 (EC_{50} OF THE ΣF). GREEN COLOURED BARS REFER TO NON-TOXIC FRACTIONS. ERROR BARS CORRESPOND TO THE STANDARD DEVIATION (SD) OF THE THREE REPLICATES. 10
FIGURE S6. EXAMPLE OF THE CONFIRMATION VIA SUSPECT SCREENING OF THE PHARMACEUTICAL BISOPROLOL, TOP PRIORITY COMPOUND FOUND IN THE ACTIVE FRACTIONS F6 AND F7. A) EXTRACTED ION CHROMATOGRAPH AND B) MASS SPECTRA SIMILARITY WITH THE MZCLOUD LIBRARY. 11
FIGURE S7. RESULTS OF SEA URCHIN *P. LIVIDUS* LARVAE EXPOSED TO REF 50 OF INDIVIDUAL COMPOUNDS DETECTED IN THE TOXIC FRACTIONS USING THE SETAPP [40]: A) SIZE INCREASE (%) RESPONSE. ERROR BARS CORRESPOND TO THE STANDARD DEVIATION (SD) OF THE THREE REPLICATES. B) IT VALUES BASED ON MALFORMATION OF THE OBSERVED LARVAE. 12
FIGURE S8. RESULTS OF SEA URCHIN *P. LIVIDUS* LARVAE EXPOSED TO DIFFERENT CONCENTRATIONS OF THE ARTIFICIAL MIXTURE CONTAINING TOXICITY DRIVER CANDIDATES USING THE SETAPP [40]: A) SIZE INCREASE (%) RESPONSE. ERROR BARS CORRESPOND TO THE STANDARD DEVIATION (SD) OF THE THREE REPLICATES. B) IT VALUES BASED ON MALFORMATION OF THE OBSERVED LARVAE. C) MALFORMATION OF THE OBSERVED LARVAE: 1) REF 0.01, 2) REF 0.1, 3) REF 1, 4) REF 10, 5) REF 50 AND 6) REF 1000. 13
S5. REFERENCES .... ERROR! BOOKMARK NOT DEFINED.

S1. Reagents and materials

Compounds names, use, CAS numbers, molecular formulas and brands can be found in Lopez-Herguedas et al. [41]. All the standards used in this study have a purity of at least 95 %.

Stock standard solutions were prepared in different solvents including methanol (MeOH, UHPLC-MS quality, Scharlab, Barcelona, Spain), Milli-Q water (<0,05 S/cm, Milli-Q, 185 model, Millipore, Bedford, MA, USA), acetonitrile (ACN, ChromAR HPLC, Macron Fine Chemicals, Józefów, Poland), acetone (ChromAR HPLC), ethanol (EtOH, ChromAR HPLC) or dimethylsulfoxide (DMSO, Applichem, Panreac, Darmstadt, Germany), depending on the solubility of the compounds, in order to prepare solutions with approximately 1000-5000 mg/kg concentrations. In the particular case of methotrexate, 10 µL of NaOH (98 %, Panreac, Barcelona, Spain) was added to the solution. From these concentrated solutions, 100 and 50

mg/kg solutions of different groups of compounds were prepared, and a dilution at lower concentrations (2 mg/kg) containing all analytes was prepared before every analysis in UHPLC-MS MeOH. All the chemical standards solutions were stored at -20 °C.

Reverse-phase Chromabond HR-X (85 µm, 55-65 Å, Macherey-Nagel, Düren, Germany), and weak anion and cation exchange phases, Sepra ZT-WAX (Phenomenex, California, USA, 30 µm, 85 Å) and Sepra ZT-WCX (Phenomenex, California, USA, 30 µm, 85 Å), respectively, bulk sorbents were used for sampling and sample preparation. LV-SPE extraction cartridges (10 g, polyvinylidene fluoride, PVDF) were purchased from MAXX Mess-u. Probenahmetechnik GmbH (Rangendingen, Germany). Empty solid-phase extraction (SPE) tubes (6 mL and 20 mL) and polypropylene (PP) frits were purchased from Supelco (Bellefonte, PA, USA).

Formic acid (HCOOH, > 98 %) was supplied by Panreac (Barcelona, Spain) and used in the SPE step for compound elution. MeOH (HPLC grade, 99.9 %), ethyl acetate (EtOAc, HPLC grade, 99.9 %), acetone (HPLC grade, 99.9 %) and ammonium solution (25 % as NH_3), used in the target compounds SPE-elution, were obtained from Sigma Aldrich (St. Louis, MO, USA). HCOOH, water and ACN (UHPLC-MS grade) used as mobile phases in the LC-HRMS were provided by Fischer Scientific (Geel, Belgium).

S2. Effect-Directed Analysis

S2.1. Sampling and Sample Treatment

The aqueous sample was directly collected from the sewage system of a hospital located in Biscay, which discharges the residual waters in Galindo WWTP and subsequently releases it into Nerbioi-Ibaizabal estuary. With over 800 beds and more than 13 specialities in addition to various general services, it is one of the largest hospitals in the Basque Country. Hospital waters were collected with the LV-SPE device, equipped with a Sartopure GF+ MidiCap pre-filter (Sartorius, Gottingen, Germany) for suspended particulate matter removal. The cartridges used in the LV-SPE system were prepared in-house by filling an empty PTFE column (MAXX Mess-u. Probenahmetechnik GmbH, Rangendingen, Germany) with 6 g Strata HR-X (top), 2 g of both Strata ZT-WAX and ZT-WCX (bottom). Previous to the sampling, the cartridges were assembled, filled with the solid phase sorbents and conditioned using 200 mL of MeOH:EtOAc (50:50, v:v) and 200 mL of Milli-Q water. Then a hospital effluent aqueous sample was pumped into the LV-SPE device in 3 min cycles/h during 24 h (20 cycles of ≈ 100 mL each per hour). After sampling, each cartridge was connected to a nitrogen gas stream for ≈1 h to purge residual water. Elution was carried out with 300 mL of MeOH:EtOAc (50:50, v:v) solvent mixture with 2 % NH_3 (v/v) followed by 300 mL of MeOH:EtOAc (50:50, v:v) solvent mixture with 1.7 %

HCOOH. The extracts were combined and evaporated using rotary evaporation (Büchi, Flawil, Switzerland) and adjusted to a final volume of 180 mL (i.e. the raw sample with an enrichment factor of 250, EF 250).

S2.2. Fractionation and solid-phase extraction (SPE)

Aliquots of 250 μL of the raw sample and the procedural blank (EF 250) were fractionated by reverse phase-high performance liquid chromatography (RP-HPLC) using a gradient elution consisting of Milli-Q water and methanol (MeOH), both containing 0.1 % of formic acid (HCOOH) at a flow rate of 2.36 mL/min. The HPLC was operated under the control of Chemstation B.04.03 software and was comprised of an Agilent 1100 series HPLC chromatogram equipped with a DAD detector, an automatic autosampler and an automatic fraction collector (Agilent Technologies, series 1100, Avondale, PA, USA).

The first fractionation was performed with an RP semi-preparative C_{18} column (Macherey-Nagel Nucleodur C_{18} column, 250 x 10 mm, 5 μm particle size). The gradient started at 30 % of MeOH, held for 5 min, linearly increasing to 95 % of MeOH within 30 min and maintained for the next 15 min before returning to the initial conditions for 15 min re-equilibration. In total, 18 sample fractions (F1-F18) of two (F11-F17), three (F3-F10) and four minutes (F1, F2 and F18) intervals were collected. The second fractionation of the toxic fractions found in the first fractionation was performed using an aminopropyl (AP) column (Imtakt, 150 x 10 mm, 3 μm). The separation in this case was carried out using a gradient that started at 5 % of MeOH for 2 min and continued increasing gradually to 2.5 % each min during the next 36 min when it maintains constant at 95 %. After this period, the proportion of MeOH started to decrease to the initial conditions for a 15 min re-equilibration phase. In total, 16 fractions were collected in intervals of three min (ΣF-1-14) and four min (ΣF-15 and ΣF-16).

The fractionation step was equally conducted for the hospital effluent sample (raw) and blank sample (LV-B). The recombined fraction (RS) from the raw sample and the blank (LV-B-R) were constituted from equal volumes of all fractions collected in each fractionation and further evaluated in the same way as the individual fractions.

The solvent of all the fractions (RS and LV-B-R) was changed in order to get suitable media for SET bioassays and non-targeted analysis. To that aim, all the fractions were evaporated until the percentage of MeOH was less than 5 % in water [26] using a rotary evaporator (rotavap) system (LABOROTA 4000, Heidolph, Schwabach, Germany) and submitted to an SPE protocol [4].

The cartridges were prepared with Chromabond HR-X:Sepra ZT-WAX:Sepra ZT-WCX 3:1:1 500 mg in total (300:100:100, m:m, conditioned with 10 mL of MeOH:EtOAc (1:1, v:v) and 10 mL of Milli-Q water). The loaded cartridges were dried and eluted with 12 mL of MeOH:EtOAc (1:1, v:v) containing 2 % NH_3 (v/v) and 12 mL of MeOH:EtOAc (1:1, v:v) containing 1.7 % (v/v) HCOOH. The extracts were evaporated to ≈100 μL under a gentle stream of nitrogen at 40 °C using a TurboVap LV (Zymark, Hopkinton, EEUU) and were kept in the fridge at 4°C.

S2.3. Sea Urchin Embryo Test (SET) bioassay

Gametes were obtained by osmotic-shock-induced spawning by injecting 1 mL of potassium chloride (KCl, 0.5 M) into the peri-oral side[4]. Eggs were filtrated over 200 first and then 60 μm and were kept in falcons with seawater. Sperm was kept dry in sterilized Eppendorf tubes.

In the fertilization procedure, a few non-diluted sperm μL were added to the egg solution in a falcon tube [81]. Afterwards, polyspermy, egg density (eggs/mL) and fertilization rate (%) were checked under an inverted microscope (Nikon Eclipse Ti-S). Eggs were counted using a Sedgewick-rafter counting cell (Pyser Optics, Edenbridge, United Kingdom). Fertilized gametes were used for the bioassay only when fulfilling: no polyspermy, egg density enough to obtain a final concentration in all the tested vials of 40 eggs/mL and > 90% fertilization rate.

For the calculation of IT, 100 individual embryos were categorised for their malformation level according to Carballeira et al. toxicity category [79]. Non-toxic or Level 0 toxicity corresponds to larvae at the four-arm pluteus stage with fully developed arms, complete skeletal rods and of similar size to control larvae. Slightly toxic or Level 1 toxicity was characterized by larvae presenting an incorrect location of skeletal rods. Moderate toxicity or Level 2 corresponds to larvae with no skeleton or anomalous shape. Highly toxic or Level 3 toxicity was featured by non-developed larvae at early stages which do not reach the pluteus stage. Then, the general IT was calculated according to Equation (1):

$$IT = \frac{(0 \times \%Level\ 0) + (1 \times \%Level\ 1) + (2 \times \%Level\ 2) + (3 \times \%Level\ 3)}{100} \quad \textbf{\textit{(1)}}$$

Where, IT ranges from 0 (no toxicity) to 3 (highly toxic).

The larvae size increase was recorded according to Saco-Álvarez et al. [78] (Figure S1). The maximum dimension of 40 embryos (either normal or abnormal) was measured and the size increase was calculated by subtracting the fertilized egg diameter at t=0 (fertilized egg fixed at the initial time). The criteria of a minimum mean size of all controls (218 μm, larvae growth) with respect to the egg or the length of control larvae to be ≥ 340 μm must be fulfilled.

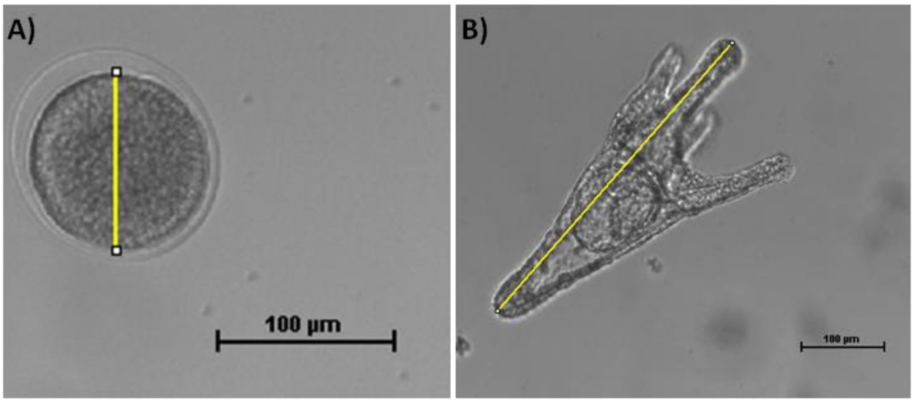

Figure S1. Examples of how to measure the maximum dimension according to Saco-Álvarez [78] in *P. lividus* at fertilized eggs (A) and completely developed larvae at 4 arm plateaus stage (B).

S2.4. Chemical analysis and data treatment

S2.4.1. LC-qOrbitrap analyses

Concerning the liquid chromatography, 5-µL aliquots of the extracts were injected on a Kinetex XB-C18 (2.1 mm x 150 mm, 2.6 µm) column with a pre-filter (2.1 mm ID, 0.2 µm) from Phenomenex (Madrid, Spain). For positive ionisation mode, a mobile phase consisting of Milli-Q water (solvent A) and ACN (solvent B), both containing 0.1 % HCOOH was used. In the negative mode, 5 mM ammonium acetate was added to both solvents. The LC gradient started at 87 % A with a linear increase to 50 % A at 5 min, and another linear change to 5 % A at 3 min (hold 3 min). Then, the gradient was returned to initial conditions in 3 min and kept for another 2 min lapse, with a flow rate set to 0.3 mL/min and a column temperature of 35°C. Measurements were performed in negative and positive ionization modes in Full scan – data-dependent MS2 (Full MS-ddMS2) discovery acquisition method in the *m/z* 70-1050 Da range. After a complete scan at a 70000 full width at half maximum (FWHM) resolution of *m/z* 200, three scans were performed in the *m/z* 100-600 Da range at 17500 FWHM at *m/z* 200 with an isolation window of 3.0 *m/z* with stepped normalized collision energy (NCE) of 10-45-90. The ddMS2 scans were acquired with an automatic intensity threshold and dynamic exclusion. ACG target was set at 5e4 and its minimum was set at 8.00e3. With respect to the HESI parameters, spray voltage was set at 3.2 kV for positive and 3.5 kV for negative ionization modes. For positive ionization, the capillary temperature was set at 320 °C, the sheath gas at 40 arbitrary units (au), the auxiliary gas at 15 au and 310 °C and the sweep gas at 1 au. For negative ionization, the capillary temperature was set at 300 °C, the sheath gas at 40 au, the auxiliary gas at 15 au and 280 °C and the sweep gas at 1 au. Xcalibur 3.1 (Thermo Fischer Scientific) software was used for instrument control.

For the suspect analysis of LC-qOrbitrap measurements, the Compound Discoverer 3.1 (CD, Thermo-Fisher Scientific) software was used. Only peaks following a Lorentzian shape and included in the NORMAN database (40,059 compounds, http://www.norman-network.net/) were considered for peak picking. Several criteria including minimum area (7.5 x10^5 and 5.0 x10^5 for positive and negative ionization, respectively), a maximum threshold of 10 for background contamination, a mass tolerance of 5 ppm for the m/z values and ≥ 30 % for the intensity tolerance for the isotope search were applied for candidate identification. Only those peaks with available MS2 spectra and relative standard deviation (RSD %) of injection replicates (n=3) lower than 30 % were considered. Moreover, regarding the proposed CD molecular formulas, only the ones explaining satisfactorily the MS1 spectra (Sfit > 30 % and pattern coverage > 80 %) were chosen. Chemical structures were assigned based on ddMS2 fragments annotated by CD. Afterwards, characteristics including the exact mass, the isotopic pattern, MS2 fragmentation and abundances of the selected features were compared with those available in the mzCloud (best match > 70 %) library (https://www.mzcloud.org/). Fragmentation was also evaluated with mzLogic tool (Thermo-Fischer Scientific) when those fragmentation spectra could explain > 50 % of the main fragments. In the case of having available standards, a ± 0.2 min of retention time was set for positive confirmation, whereas an estimated retention time (tR) from Retention Time Indices Platform (RTI, National and Kapodistrian University of Athens, http://rti.chem.uoa.gr/) was calculated for the rest. Finally, identification criteria according to Schymanski et al. [45] was noted providing the candidates with a tentative code from 1 to 4 of identification.

Target analysis of compounds identified as level 1 was performed using The TraceFinder 4.1 (Thermo-Fisher Scientific) software. In this case, the maximum error of 5 ppm was admitted in the monoisotopic mass and fragmentation mass, whereas, in the case of the t_R, a 60 s range was admitted. Lastly, the criterion of obtaining a pass in isotopic profile (IP), fragmentation (FI) and t_R (PK) was essential for peak identification. Thus, the similarity of the theoretical and experimental IP greater than 70 % was considered. Compounds not fulfilling those conditions were rejected.

S2.4.2 GC-MS analysis

2µL-aliquots of the extracts were also analysed by GC-MS. The analysis was run in a splitless mode for 1.5 min at 250 °C using a DB-WAX (30 m × 0.25 mm, 0.25 µm, Agilent) column. The oven temperature was programmed as follows: start at 60 °C for 1 min, increase at 20 °C/min to 250 °C and held at 250 °C for 60 min. Helium (Air liquid, 99.9995 %, Madrid, Spain) was used as carrier gas at a constant flow of 1.3 mL/min. The MS transfer line, ion source and quadrupole

analyser temperatures were maintained at 300, 230 and 150 °C, respectively, and a solvent delay of 3 min was selected. Measurements were carried out using electron impact ionisation at the scan mode (*m/z* 50 – 525) to first detect and identify unknown compounds. Once the analytes were identified through NIST library, their presence in the samples was confirmed by the use of standards and quantified in the SIM mode.

The Chemstation (Agilent) software was used for data treatment. The similarity of the theoretical and experimental mass spectra greater than 90 % was considered based on the NIST17 MS library. In the case of the t_R, a 0.02 min range was admitted. The ions monitored (SIM mode) for diethyl phthalate and the 2,4-di-tert-butylphenol were 149/177 and 191/206, respectively.

S3. EDA-Quality Control

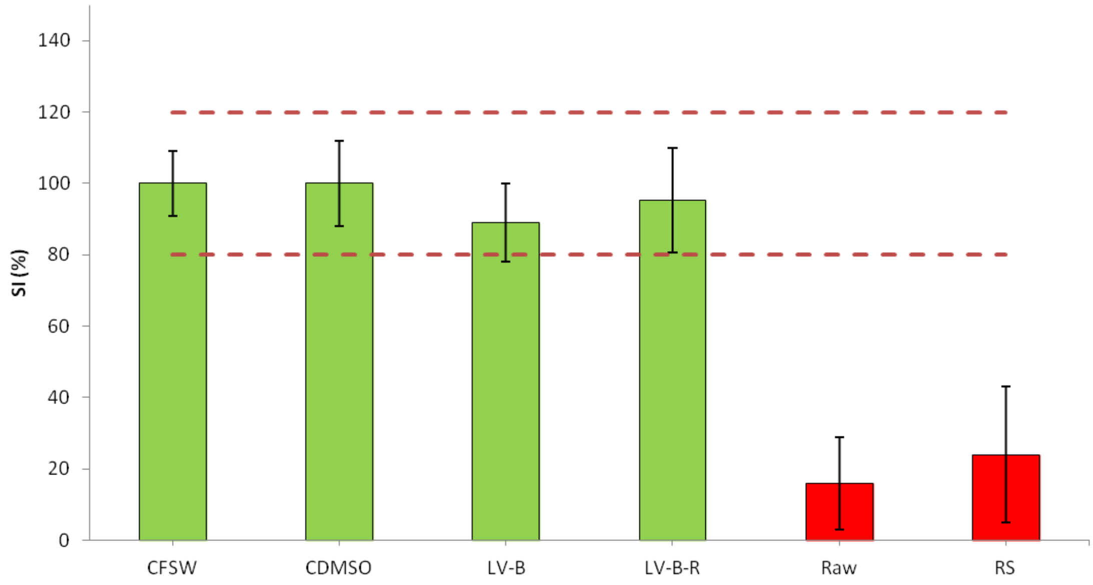

Figure S2. Toxicity of the tested procedural blanks (LV-Blank and LV-R-Blank) and the difference with the Raw and RS samples considering only the SI (%) endpoint. A comparison with solvent controls (CFSW and CDMSO) is also included. Green coloured bars refer to non-toxic samples/concentrations, while red bars refer to toxic samples/concentrations. Error bars correspond to the standard deviation (SD) of the three replicates.

S4. EDA-SET

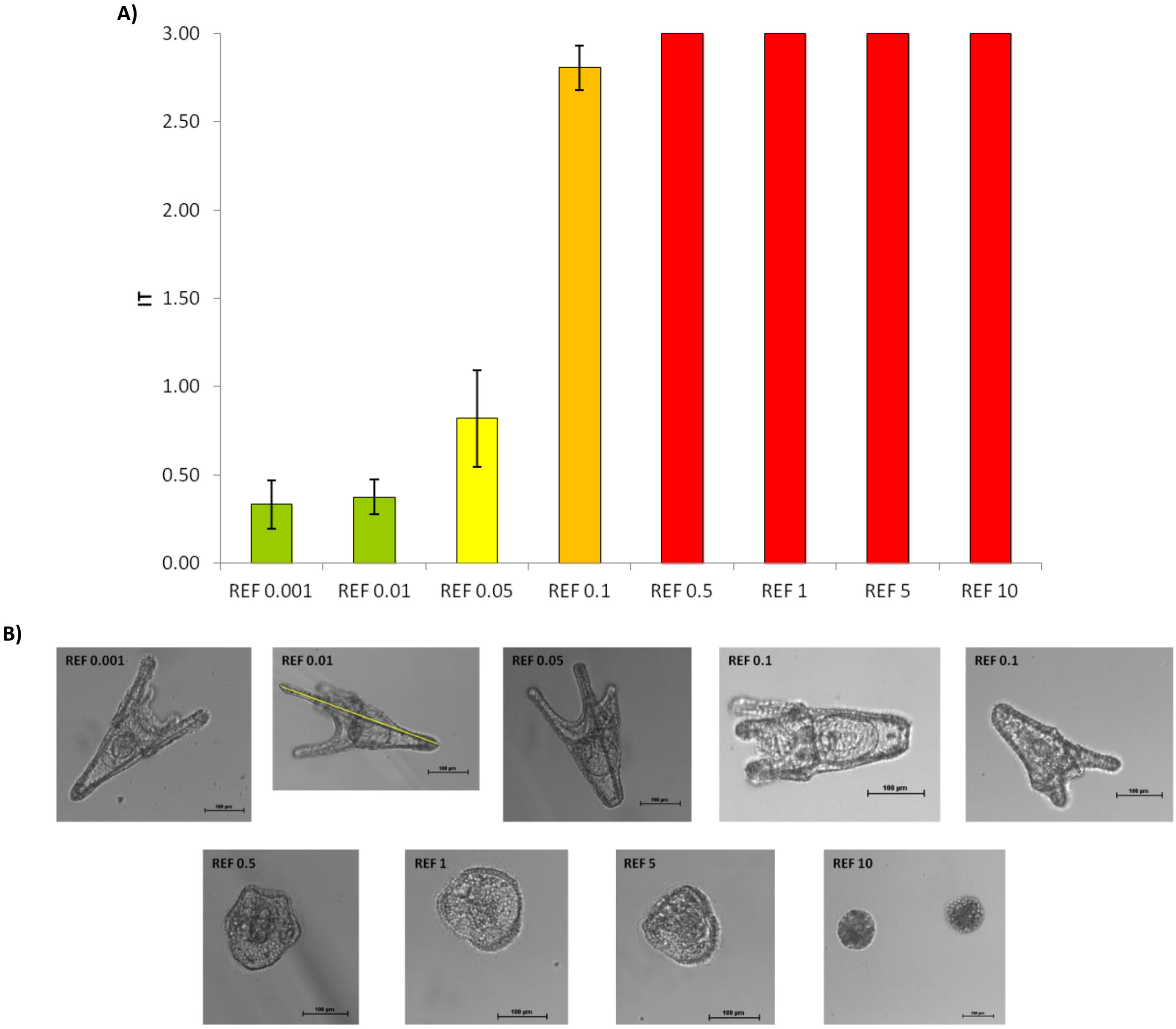

Figure S3. Toxicity level measured for each REF of the hospital effluent according to larvae malformation endpoint: A) Calculated Index of Toxicity (IT) for each REF of the sample. Colour bar shift goes from non-toxic REFs (green) to REFs with higher toxicity (red). Error bars correspond to the standard deviation (SD) of the three replicates. B) Malformation of the observed larvae in each REF of the sample.

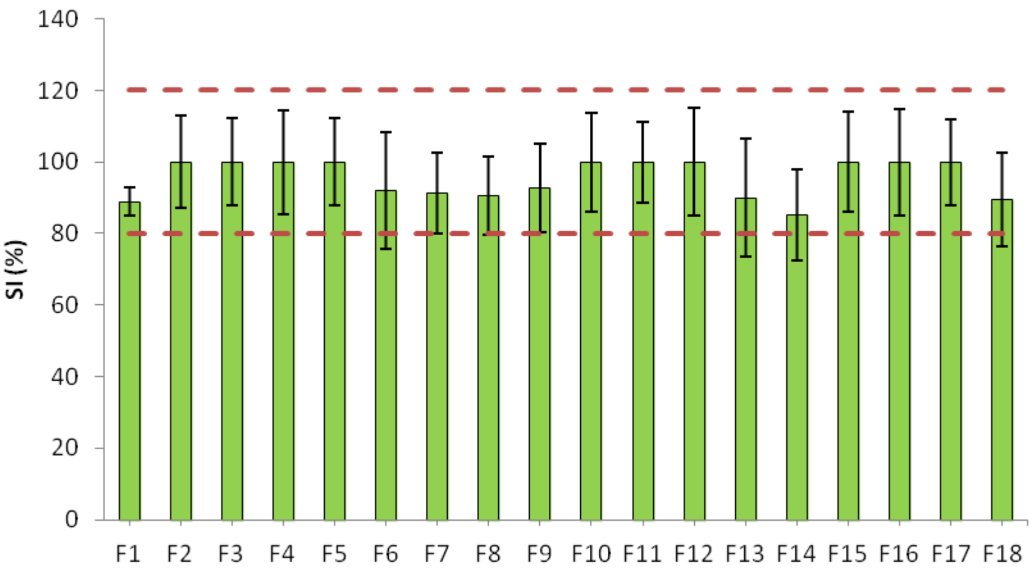

Figure S4. Size increase (%) response of the fractions from the first fractionation at REF 0.07 (EC_{50} of the raw sample). Green coloured bars refer to non-toxic fractions. Error bars correspond to the standard deviation (SD) of the three replicates.

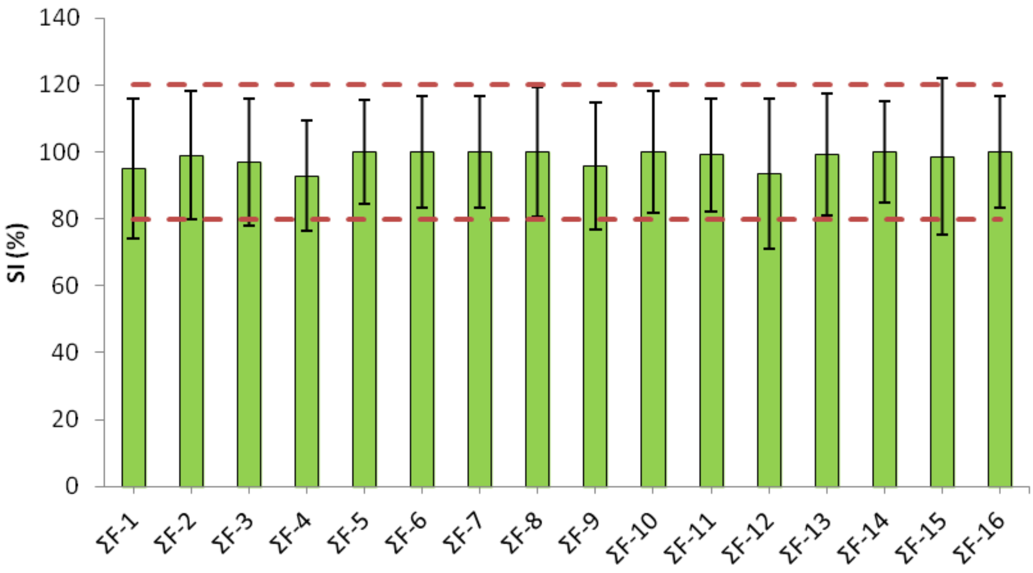

Figure S5. Size increase (%) response of the fractions from the second fractionation at REF 0.5 (EC_{50} of the ΣF). Green coloured bars refer to non-toxic fractions. Error bars correspond to the standard deviation (SD) of the three replicates.

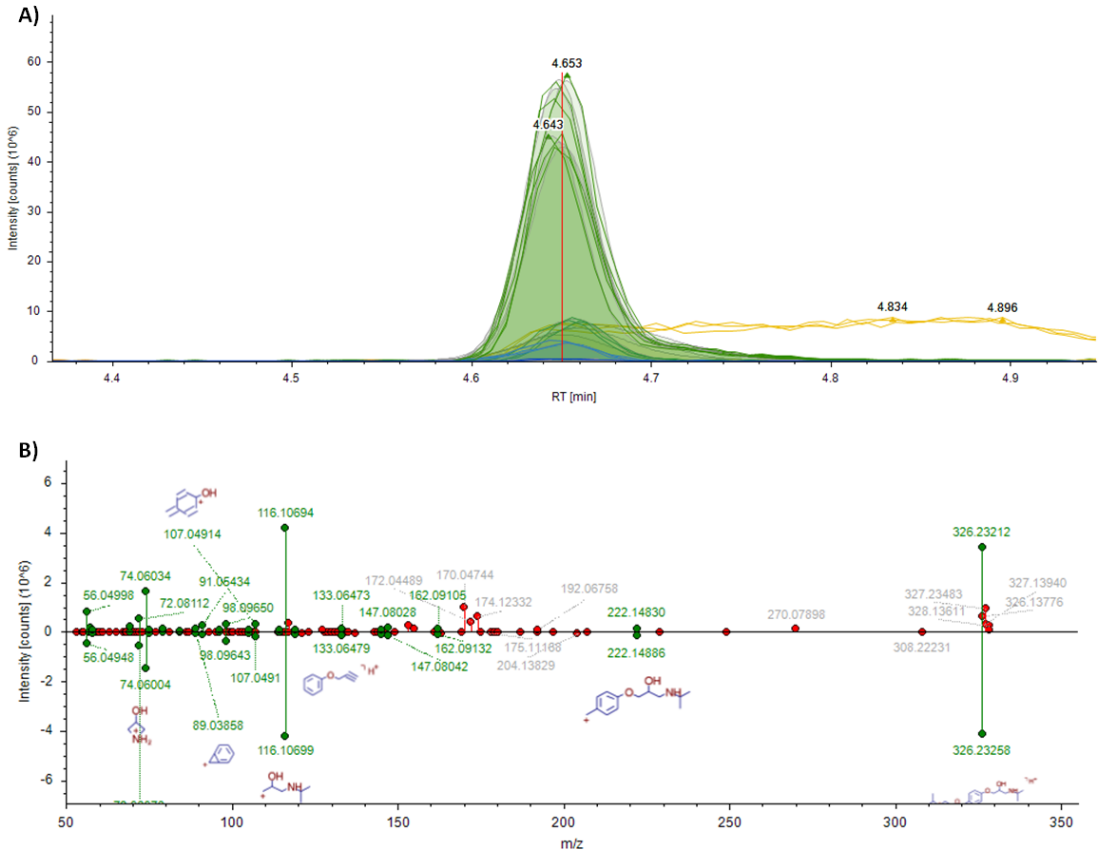

Figure S6. Example of the confirmation via suspect screening of the pharmaceutical bisoprolol (level 1), top priority compound found in the active fractions F6 and F7. A) Extracted ion chromatogram and B) experimental and mzCloud library mass spectra similarity.

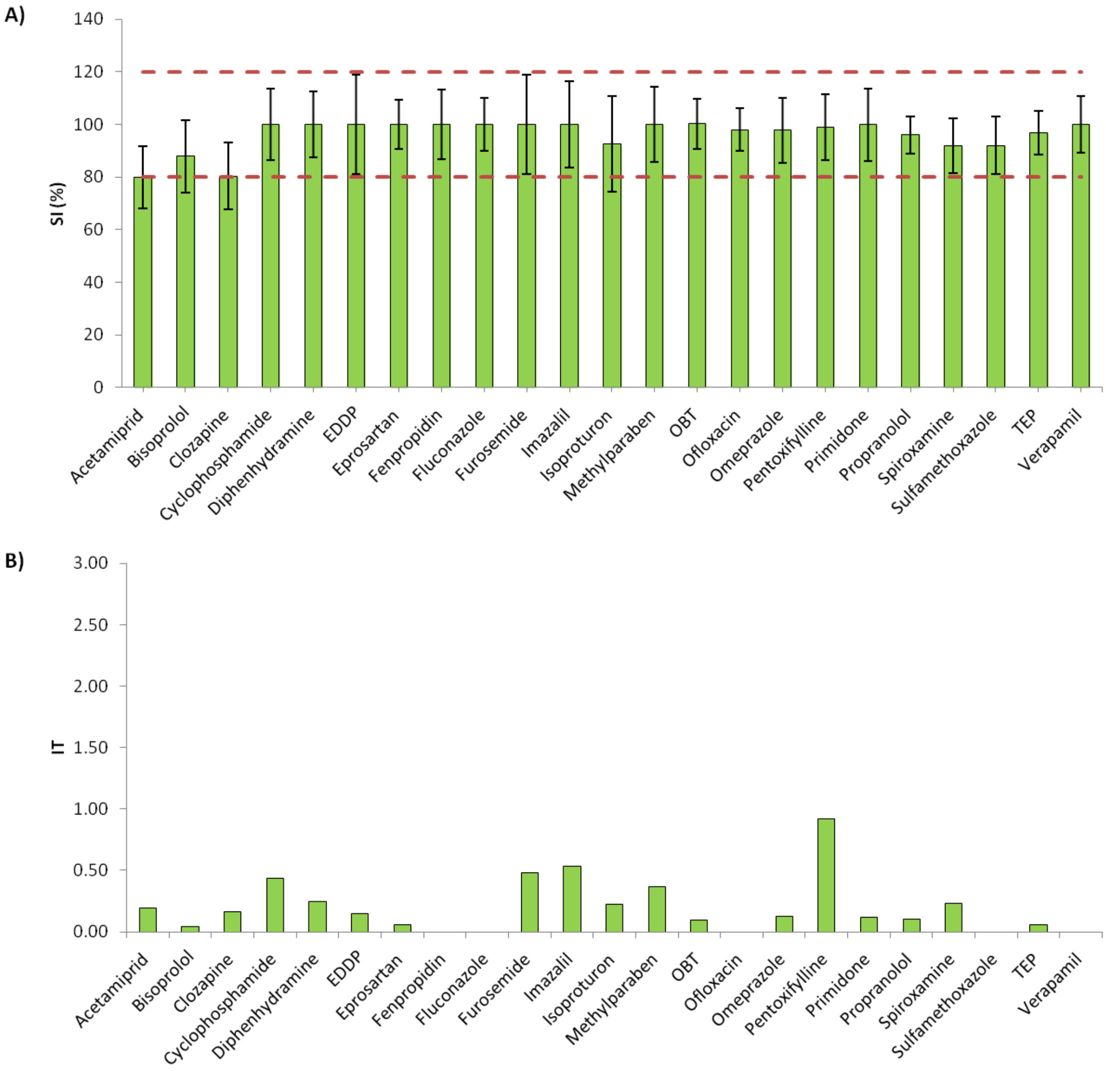

Figure S7. Results of sea urchin *P. lividus* larvae exposed to REF 50 of individual compounds detected in the toxic fractions using the SETApp [40]: A) Size increase (%) response. Error bars correspond to the standard deviation (SD) of the three replicates. B) IT values based on malformation of the observed larvae.

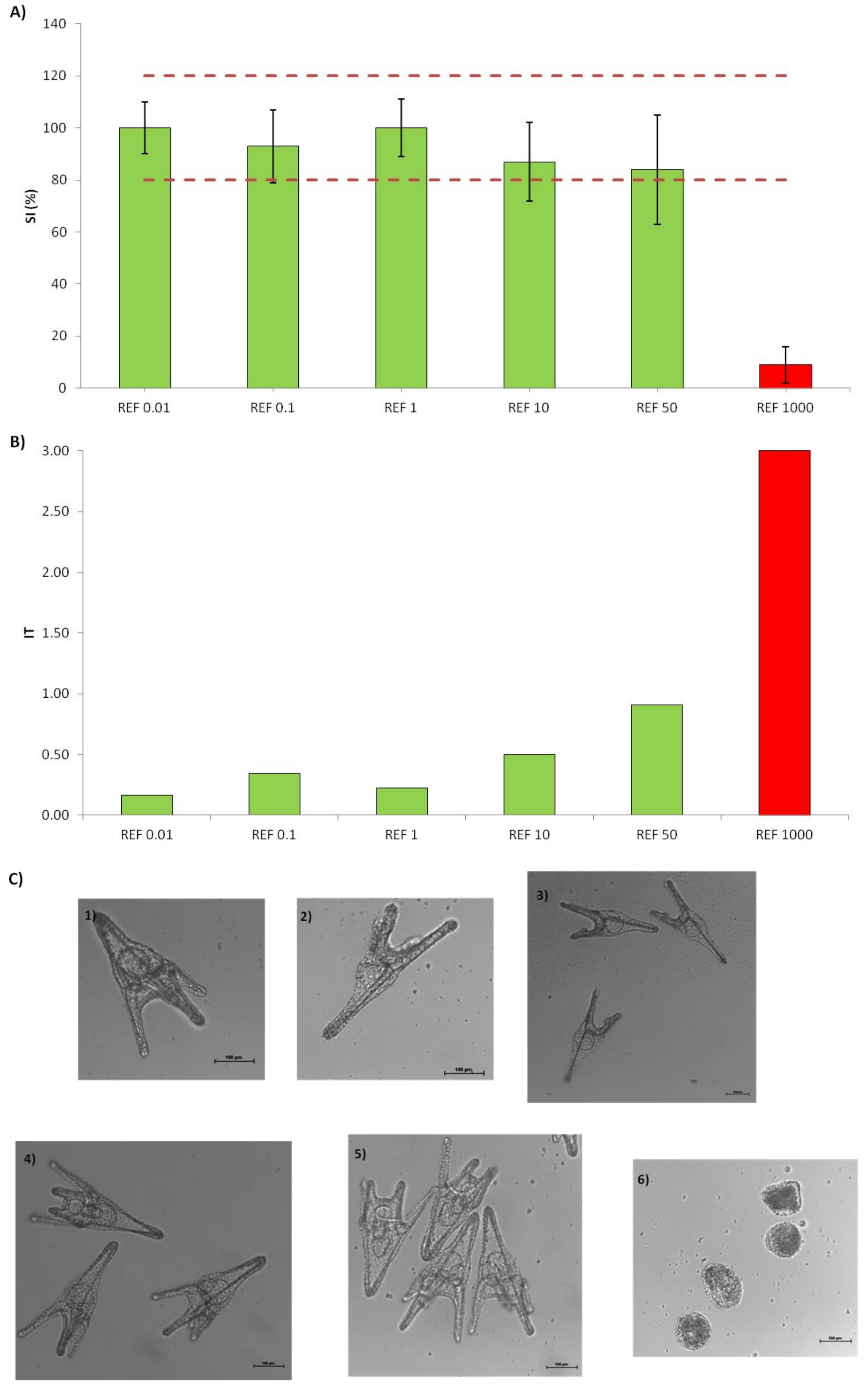

Figure S8. Results of sea urchin *P. lividus* larvae exposed to different concentrations of the artificial mixture containing toxicity driver candidates using the SETApp [40]: A) Size increase (%) response. Error bars correspond to the standard deviation (SD) of the three replicates. B) IT values based on malformation of the observed larvae. C) Malformation of the observed larvae: 1) REF 0.01, 2) REF 0.1, 3) REF 1, 4) REF 10, 5) REF 50 and 6) REF 1000.
